# Knowledge, attitudes and practices assessment of malaria interventions in rural Zambia

**DOI:** 10.1186/s12889-020-8235-6

**Published:** 2020-02-12

**Authors:** Desmond T. Jumbam, Jennifer C. Stevenson, Japhet Matoba, John P. Grieco, Lacey N. Ahern, Busiku Hamainza, Chadwick H. Sikaala, Pascalina Chanda-Kapata, Esther I. Cardol, Passwell Munachoonga, Nicole L. Achee

**Affiliations:** 1 Department of Biological Sciences, Eck Institute for Global Health, University of Notre Dame, Indiana, USA; 2 Johns Hopkins Malaria Research Institute, Johns Hopkins Bloomberg School of Public Health, Baltimore, USA; 3 Macha Research Trust, Choma, Zambia; 4 National Malaria Control Centre, Ministry of Health, Lusaka, Zambia; 5 Ministry of Health, Lusaka, Zambia; 6 Radboud University, Nijmegen, Netherlands

**Keywords:** Malaria, Knowledge, Attitudes, And practices (KAP), Zambia, ITN, IRS, Spatial repellents

## Abstract

**Background:**

Despite rapid upscale of insecticide-treated nets (ITNs) and indoor residual spraying (IRS), malaria remains a major source of morbidity and mortality in Zambia. Uptake and utilization of these and novel interventions are often affected by knowledge, attitudes and practices (KAP) amongst persons living in malaria-endemic areas. The aims of this study were to assess malaria KAP of primary caregivers and explore trends in relation to ITN use, IRS acceptance and mosquito density in two endemic communities in Luangwa and Nyimba districts, Zambia.

**Methods:**

A cohort of 75 primary caregivers were assessed using a cross-sectional, forced-choice malaria KAP survey on ITN use, IRS acceptance and initial perception of a novel spatial repellent (SR) product under investigation. Entomological sampling was performed in participant homes using CDC Miniature Light Traps to relate indoor mosquito density with participant responses.

**Results:**

Ninety-nine percent of participants cited bites of infected mosquitoes as the route of malaria transmission although other routes were also reported including drinking dirty water (64%) and eating contaminated food (63%). All caregivers agreed that malaria was a life-threatening disease with the majority of caregivers having received malaria information from health centers (86%) and community health workers (51%). Cumulatively, self-reported mosquito net use was 67%. Respondents reportedly liked the SR prototype product but improvements on color, shape and size were suggested. Overall, 398 mosquitoes were captured from light-trap collections, including 49 anophelines and 349 culicines. Insecticide treated nets use was higher in households from which at least one mosquito was captured.

**Conclusions:**

The current study identified misconceptions in malaria transmission among primary caregivers indicating remaining knowledge gaps in educational campaigns. Participant responses also indicated a misalignment between a low perception of IRS efficacy and high stated acceptance of IRS, which should be further examined to better understand uptake and sustainability of other vector control strategies. While ITNs were found to be used in study households, misperceptions between presence of mosquitoes and bite protection practices did exist. This study highlights the importance of knowledge attitudes and practice surveys, with integration of entomological sampling, to better guide malaria vector control product development, strategy acceptance and compliance within endemic communities.

## Background

Malaria is one of many arthropod-borne diseases that contributes significantly to the global health burden. In 2017, there were an estimated 219 million malaria cases and 435,000 malaria deaths worldwide, a substantial decrease from 239 million cases and 607,000 deaths in 2010 [1]. Sustaining reductions in malaria prevalence remains a challenge in many areas in Zambia. In 2015, there were 4,077,547 reported cases of malaria and 3257 deaths resulting from malaria in Zambia [2]. The 2015 Zambia Malaria Indicator Survey (MIS) reported a nationwide malaria parasite prevalence by microscopy of 19.4%, up from 16.0% in 2010 [3]. However severe anemia prevalence decreased during the same period from 9.0% in 2010 to 6.4% in 2015. Much of the decline in severe anemia suggest reductions in chronic infection of malaria. This may primarily be due to the rapid scale-up of vector control interventions like insecticide-treated nets (ITNs) and indoor residual spraying (IRS). The National Malaria Elimination Programme (NMEP) aims to attain universal coverage of ITNs with all sleeping spaces in all households covered by an ITN [3]. As of 2015, the national average for ITN coverage was 79.5% [3]. Indoor residual spraying varied significantly across provinces with the highest coverage in Eastern province (56%) and the lowest in Lusaka Province (17.4%) [3]. Despite the scale-up of ITNs and IRS, residual malaria transmission will remain a challenge to overcome.

Residual transmission, defined as persistent malaria transmission despite high coverage of ITNs and IRS [4], can result from many factors, to include mosquitoes evading current vector control strategies due to their behavior (early-evening biting, outdoor biting). This is due to varied causes such as human behavior, insecticide resistance and/or change in vector behavior [5]. An increasing number of studies report shifts of certain mosquito species to outdoor biting [6–8]. The primary vectors of malaria in Zambia are *Anopheles gambiae* sensu stricto (s.s.), *An. funestus s.s.* and *An. arabiensis* [9]*.* Resistance to pyrethroids, carbamates and DDT, a critical concern that has the potential to limit ITNs and IRS efficacy, has been reported in *An. gambiae* and *An. funestus* in Zambia to varying degrees, particularly in areas with high IRS coverage [10, 11]. Zambia has recently switched to spraying with organophosphates, specifically pirimiphos-methyl (Actellic CS) to combat this resistance. Similar challenges with insecticide resistance have also been reported in many parts of the world [12] stressing the need for new insecticides and/or innovative interventions to complement ITNs and IRS to maintain their efficacy, where they are still effective.

As ITN and IRS coverage increases, outdoor biting mosquitoes that had previously been ignored may become culpable of malaria transmission due to selective suppression of indoor-biting mosquitoes [6, 13] and other interventions will be required [4, 12]. Other documentation of change in mosquito host-seeking behavior includes shifting to early-evening and early-morning biting, when people are less likely to be using ITNs [14, 15]. These mosquito behavioral changes suggest that other interventions will be required to sustain the gains made on malaria control as well as achieve malaria elimination. Spatial repellents are one among many new vector control tools being evaluated as a complementary strategy towards combatting residual transmission.

Studies in Africa have shown that increasing knowledge and awareness of vector control tools may increase the uptake of novel malaria control interventions [16, 17]. Repellent products such as mosquito coils, topical repellent creams and spatial repellent emanators have been proven effective to protect against malaria [18–24]. The current study assessed acceptability of a spatial repellent (SR) product in development. Spatial repellents (SR) are products that release vaporized chemicals which elicit mosquito behavioral responses that result in reduction in human-vector contact [19] such as movement away from a chemical stimulus, host attraction inhibition and/or feeding inhibition [20]. Various spatial repellent products have been demonstrated to be effective against insecticide-resistant mosquitoes and outdoor-biting, day-biting and early-evening biting mosquitoes [18]. Two epidemiological trials have also indicated that spatial repellents can reduce malaria infection, suggesting a public health value. Specifically, Hill et al. in China showed a 90% decrease in *P. falciparum* and *P. vivax* infection rates when evaluating 0.03% transfluthrin mosquito coils in combination with long-lasting insecticide treated nets (LLINs) [26]. In addition, Syafruddin et al. in Indonesia indicated a 52% reduced incidence using metofluthrin coils with an associated 32% reduced human biting rate of local anopheline vectors [27]. In combination with current interventions, SRs have the potential of mitigating residual transmission in certain settings [28], although more rigorous large-scale epidemiological trials are needed to definitively endorse spatial repellents for public health use.

Until new products are available for NMEPs to deploy, ensuring the sustainability and use of ITNs and IRS is vital to maintaining their impact against malaria. This requires a better understanding of region-specific cultural and demographic factors that influence community use and acceptance of vector control interventions [29]. Low community acceptance resulting in low compliance renders interventions far less efficacious than their demonstrated or theoretical effect [30]. Knowledge, attitudes and practices (KAP) surveying is a simple common methodology that has been used to assess and identify gaps in community KAP that may influence uptake of vector control interventions [31–33]. This method is especially important for providing appropriate information for developing tailored messages and approaches in Behavior Change Communication (BCC) and education campaigns [34]. The presence or absence of mosquitoes in homes can also influence use and uptake of interventions, therefore combining entomological data with KAP surveys can provide further insight into health-seeking behavior as well as how best to implement and sustain interventions [29, 35].

The specific aims of this study were to: 1) assess caregiver KAP related to malaria transmission, disease, current and novel prevention strategies; and 2) explore response trends in relation to participant ITN use, IRS acceptance and household mosquito density. Findings generated are intended to provide guidance on educational campaigns focused on sustainability of malaria control strategies in the study districts.

## Methods

### Study design

A cross-sectional, household survey was conducted between May and June 2016 in Nyimba and Luangwa districts, Zambia. Quantitative data were collected by interviewing the primary caregiver in each household using a structured questionnaire consisting primarily of predetermined closed-ended questions and some open-ended questions. The primary caregiver was defined as the woman in the household over the age of 18 years, who was generally in charge of family care and household maintenance. Indoor host-seeking mosquito collections were performed using CDC light traps [36].

### Study site and population

**Fig. 1:**
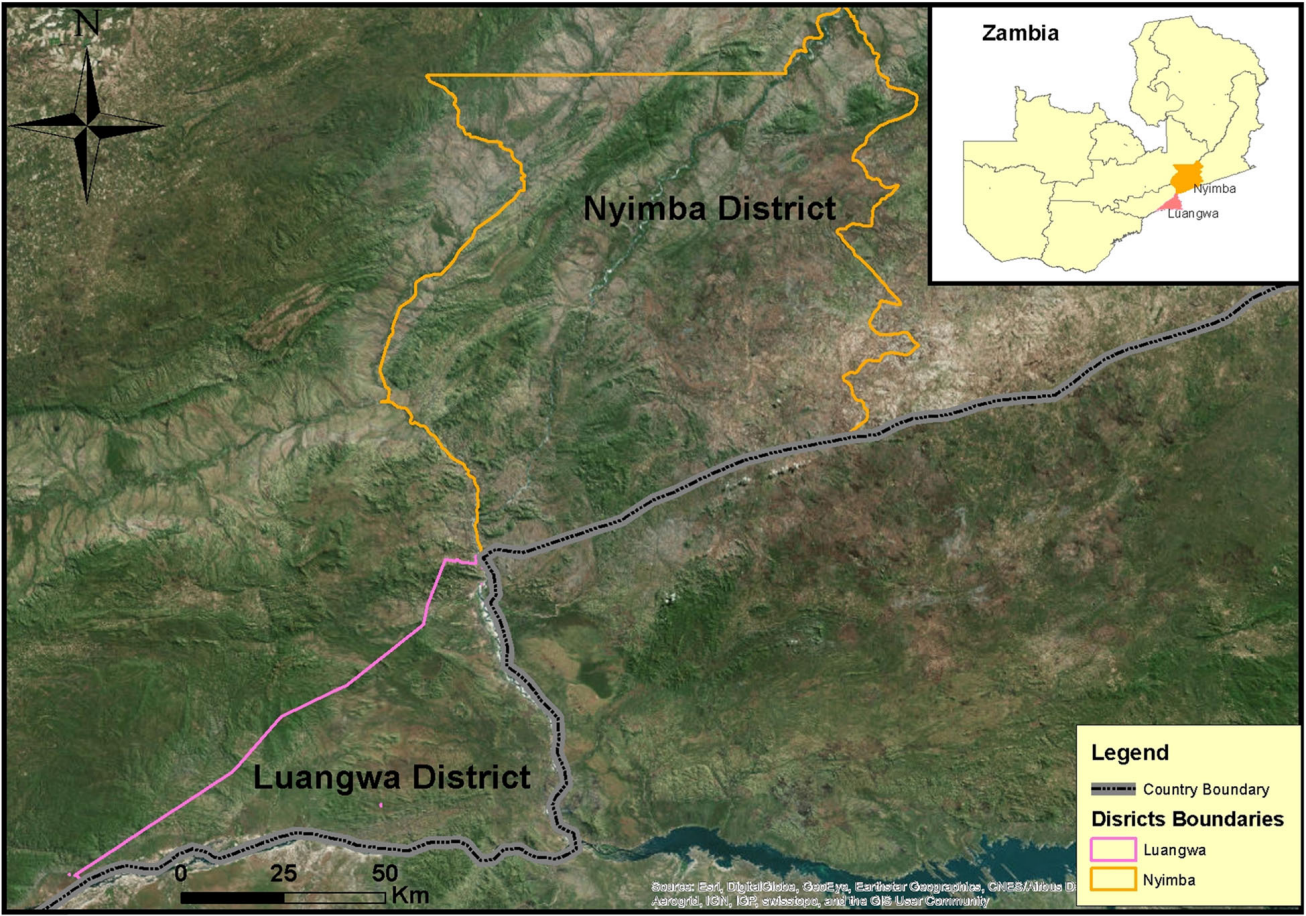
Terrain and locations of Luangwa and Nyimba Districts in Zambia. This map was created by the authors

**Fig. 2:**
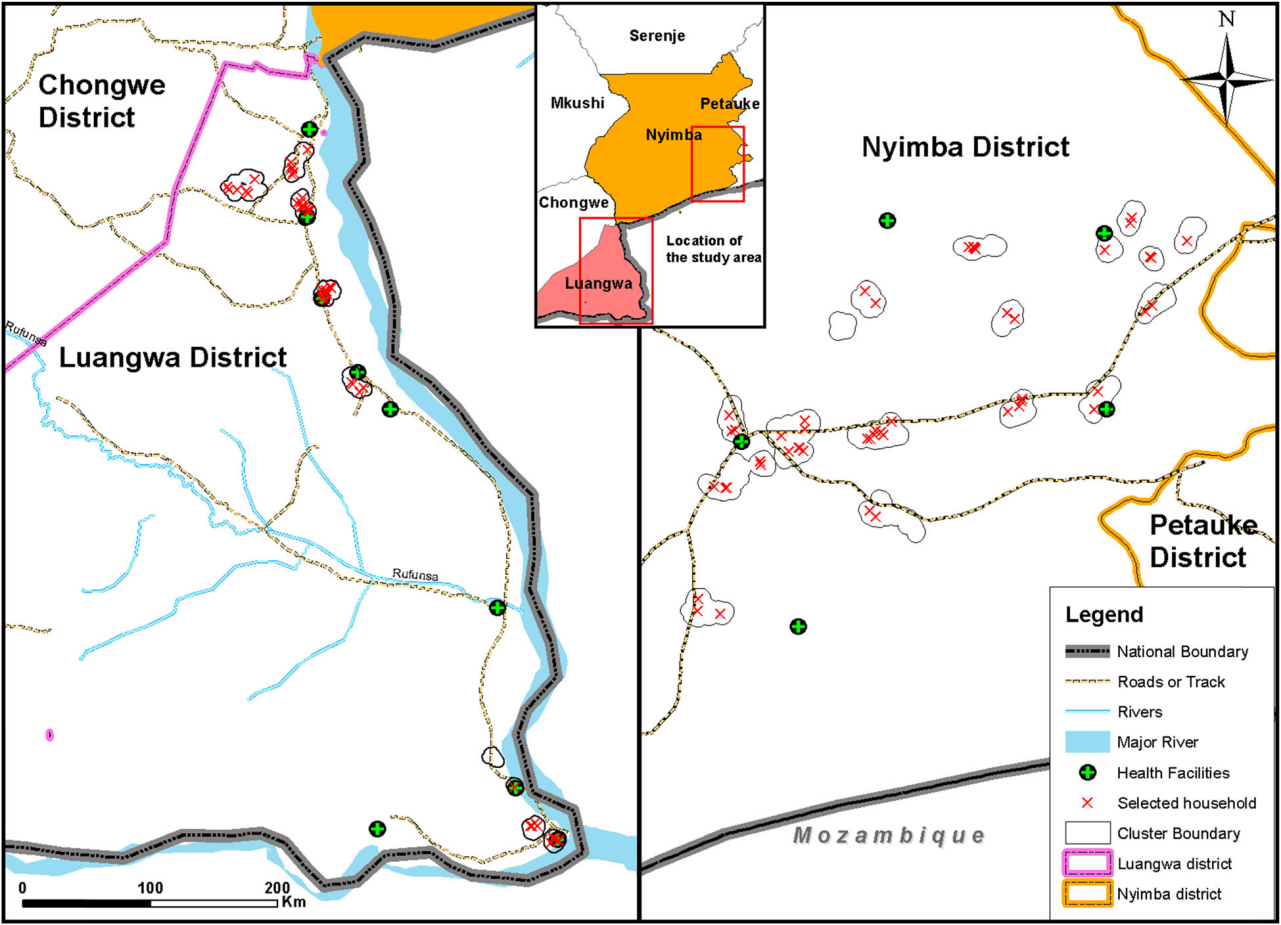
Map of Luangwa and Nyimba districts in Zambia and locations of surveyed households within each district. This map was created by the authors

Zambia is a land-locked country located in southern Africa with a population of 13,045,508 people [3]. The country is divided into 10 provinces and 105 districts. Study communities were within two neighboring malaria endemic districts, Luangwa and Nyimba districts, (Figs. 1 and 2) both classified as rural areas with the primary economic activities being fishing, agriculture and animal husbandry. Nyimba district is located in Eastern Province bordering Mozambique, with a population of 85,684 people while Luangwa district is located in the east of Lusaka Province, bordering Mozambique and Zimbabwe, with a population of 25,294 people [37]. The weather in Zambia is primarily tropical with three distinct seasons: the cool dry winter from May to August, a hot dry season from August to October and warm wet season from November to April, and a cool dry season between May and July [37]. Mosquito abundance increases after the onset of the rains in December, generally peaking in February/March. Malaria cases generally peak in April–May and falls off in June–July when rains stop [38]. Transmission of *Plasmodium falciparum* is perennial in both districts with most transmission being attributed to *An. funestus* vectors [16].

### Sampling strategy

The sampling frame consisted of households that were to be enrolled in a SR efficacy trial to include 1638 households (8 clusters) in Luangwa and 3003 households (16 clusters) in Nyimba. Clusters of households for the SR efficacy trial were chosen to meet criteria of: at least 50% of households having a child under 5 years of age, being separated from another cluster by at least 1-2 km, having approximately 200 households, and falling within the catchment area of a health facility. For this KAP study, a sample size of 75 households were chosen to ensure representation and for logistical feasibility. Systematic random sampling was used to select 40 households in Nyimba and 35 households in Luangwa, with at least one household from each cluster (Fig. 2). In cases where the house was vacant or occupants away at the time of the visit, the nearest household with an eligible participant was engaged.

### KAP surveys

KAP surveys were conducted by trained study staff and district health officers. The questionnaire was translated to Chichewa, the local language in both study districts. The survey was divided into three main sections with a total of 70 questions: 1) 27 questions related to demographic factors and knowledge; 2) 27 questions related to cultural attitudes and practices and; 3) 16 questions related to acceptability of vector control interventions (See Additional file 1). Survey questions were adapted from the Zambian Malaria Indicator Survey questionnaires [3], previously published KAP studies [8] and by the study team to include additional factors not previously captured. In each household, the KAP questionnaire was administered to one consenting female primary caregiver over the age of 18 years.

### SR product

The SR product is a late-stage development experimental product; thus not currently available on the market. For that reason we wanted to explore early, initial perceptions on design and concept. It contains transfluthrin, a volatile pyrethroid chemical currently used in household mosquito control product such as coils. Preliminary findings of a robust cluster randomized, double-blinded placebo-controlled trial in Indonesia suggests protective effects of the SR product evaluated in this study [17].

As the SR prototype was not deployed ahead of the study as originally anticipated, interviewers presented a prototype to the caregiver and gave explanations and demonstrations about how the SR was designed to work and how it would be applied in the home. Participants were given the chance to examine physical features of the prototype and ask questions of investigators. The completed surveys were checked for inconsistencies such as missing responses by another member of the study team.

### Mosquito collections and identification

Indoor mosquito collections were conducted using CDC Miniature Light Traps with incandescent lights (John W. Hock Company, Gainesville, Florida, USA) [36]. Verbal consent to set traps was sought from caregivers and/or the household head who consented to the KAP survey. A study team member positioned one trap indoors at the foot of the participant’s sleeping space (~ 1.5 m above floor level) [39] immediately after the KAP survey was completed. The battery was connected at one terminal only. Participants were shown how to connect the other terminal of the battery and instructed in local Chichewa language to start the trap at 6:00 PM that same night and tie the collection bag of the collection cup at 6:00 AM. Participants were asked to sleep under an LLIN (provided by the Ministry of Health) on the night of the collection to reduce their exposure to mosquito bites which may result from the attraction of the trap. Traps and collection bags were collected by study team members the following morning. Captured mosquitoes were killed on-site by exposure to ethanol fumes, then counted and stored in individual vials labeled by house code and date for later processing.

Identification of anophelines or culicines was conducted using morphological keys [30, 33]. The DNA from mosquito abdomens was extracted using a modified salt extraction method described by Kent & Norris [40, 41]. Molecular identification of *An. funestus s.l.* and *An. gambiae s.l.* was done by PCR as previously described by Scott et al. and Koekemoer et al. [42, 43]. All PCR products were visualized after gel electrophoresis onto a 2% agarose gel. For those samples without amplification product on either the *An. funestus* or *An. gambiae* PCR, the anopheline ribosomal DNA internal transcribed spacer 2 (ITS2) PCR assay as described elsewhere [43, 44] and modified by Das et al [45] was used to assess the identity of these mosquitoes. PCR analysis was used to detect and identify host blood from all specimens from which DNA was extracted. This multiplex PCR assay targeted the cytochrome b region of the hosts mitochondrial DNA [40]. *Plasmodium falciparum* circumsporozoite positivity was analyzed using ELISA antibody detection of head and thoraces of all female anopheline mosquitoes [46].

### Data analysis

KAP survey responses were coded then digitally entered using Microsoft Excel software and transferred to SPSS software package (SPSS version 22, Chicago, IL) for analyses at both district and household level. Categorical variables were reported using descriptive statistics such as frequencies and percentages and continuous variables reported as means, standard deviations. Variables analyzed included self-reported use and acceptance of vector control interventions, socio-demographic factors, malaria-related knowledge, attitudes and practices and indoor mosquito densities.

## Results

### Demographics of study participants

A total of 75 participants were surveyed from May–June 2016 (Table 1). About three quarters (73%, 54/75) of participants were over thirty years old and over half (55%, 41/75) had less than primary level education. The most common occupation reported by participants was farming (64%, 48/75).

**Table 1:** Caregiver^a^ (participant) demographics from Nyimba and Luangwa study Districts during the study period

Category	Nyimba *N* = 40 n (%)	Luangwa *N* = 35 n (%)	Total *N* = 75 n (%)
Age (years)			
18–30	11 (28)	10 (29)	21(28)
31+	29 (72)	25 (71)	54 (72)
Level of education of the head of the Household			
Below Primary	22 (55)	19 (54)	41 (55)
Above Primary	16 (40)	14 (40)	30 (40)
Missing/ Don’t know	2 (5)	2 (6)	4 (5)
Occupation of the head of the household			
Farming	26 (69)	22 (63)	48 (64)
Fishing	0 (0)	1 (3)	1 (1)
Business	3 (8)	1 (3)	4 (6)
Government officer	1 (3)	2 (6)	3 (4)
Driver	1 (3)	3 (9)	4 (6)
Builder	2 (5)	3 (9)	5 (7)
Other	5 (13	3 (9)	8 (11)

^a^woman > 18 years in charge of family care and household maintenance

### Knowledge of malaria disease and interventions

**Fig. 3:**
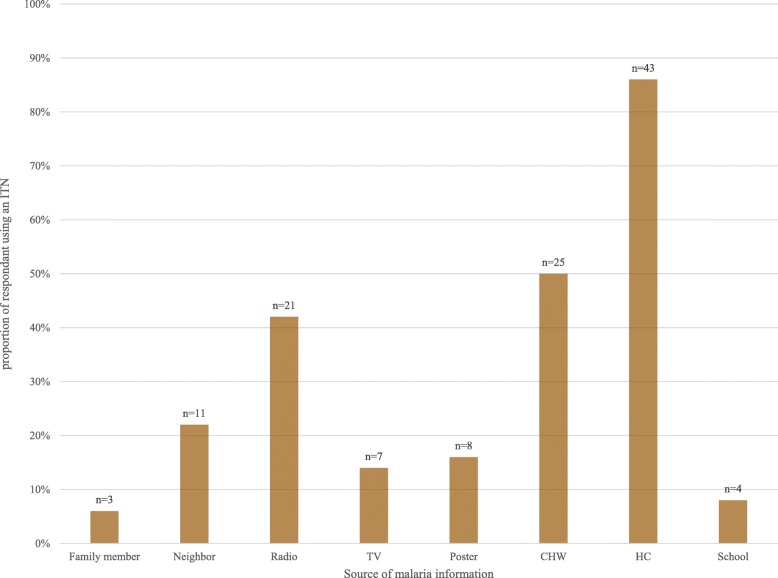
Proportion of ITN use by source of malaria information

A majority of caregivers (92%, 69/75) in both districts reported ever hearing or receiving malaria information. The most common source of malaria information reported by both districts was from the health center/clinic (49%, 37/75), followed by the community health care worker (35%, 26/75) and via the radio (39%, 29/75) (Table 2). Trend analysis showed that ITN use was highest among caregivers who received malaria information from the health center (86%, 43/50), community health worker (50%, 25/50) and radio (42%, 21/50) (Fig. 3).

**Table 2:** Caregiver^a^ (participant) reported source of malaria information in Nyimba and Luangwa Districts during the study period

Source	Nyimba n (%)	Luangwa n (%)	Total n (%)	
Family member	1 (3)	2 (6)	3 (4)	
Neighbor	12 (30)	4 (11)	16 (21)	
Radio	21 (53)	8 (23)	29 (39)	
Television	6 (15)	4 (11)	10 (13)	
Poster/information sheets	7 (18)	8 (23)	15 (20)	
Community Health Worker	22 (55)	4 (11)	26 (35)	
Health center/clinic	35 (88)	2 (6)	37 (49)	
School	1 (3)	16 (46)	17 (23)	
Other	1 (3)	1 (3)		2 (3)

Percentages may not add up to 100% because more than one source could be reported per participant

^a^woman > 18 years in charge of family care and household maintenance

#### Route of transmission and symptomology

**Fig. 4:**
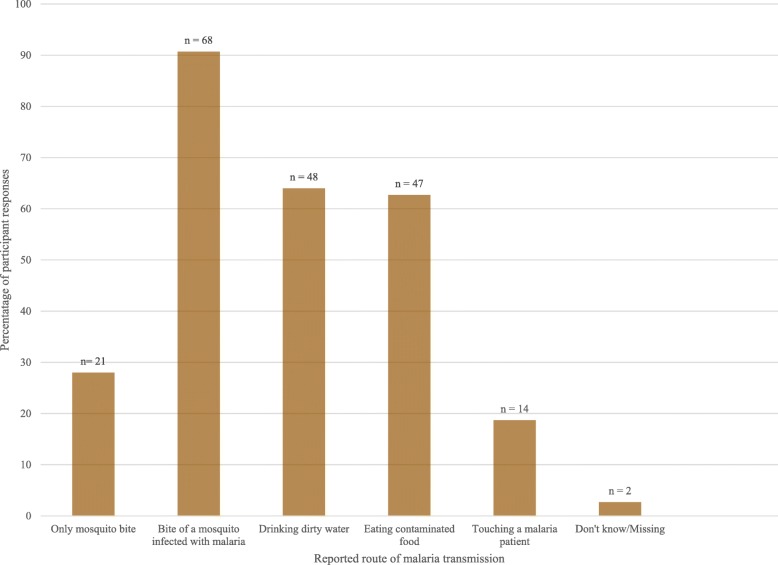
Caregiver responses related to malaria transmission route in Nyimba and Luangwa districts study districts, Zambia

When asked about the vector of malaria, all but one participant cited infected mosquitoes. In addition to this response, other commonly cited vectors included flies (43%, 32/75), cockroaches (32%, 24/75), rats (25%, 19/75) and dogs (23%, 17/75). About half (53%, 40/75) of the caregivers exclusively reported infected mosquitoes as the only vectors capable of transmitting malaria. When asked about malaria transmission routes, a majority of participants (91%, 68/75) reported that malaria could be transmitted by the bite of an infected mosquito (Fig. 4). Other transmission routes reported included drinking dirty water (64%, 48/75), eating contaminated food (63%, 47/75), and touching a malaria patient (19%, 14/75). Of the caregivers who reported malaria could be transmitted by the bite of an infected mosquito, 72% also cited other routes of transmission. Trend analysis showed that all caregivers who responded that infected mosquitoes were the vectors of malaria, reported sleeping under an ITN; however, almost all (96%, 24/25) caregivers who reported no ITN use also named the infected mosquito as the vector of malaria. Reported ITN use was 14% higher among participants who reported only the infected mosquito as the vector of malaria.

All caregivers reported high temperature/fever and headache as symptoms of malaria disease. Vomiting (97%, 73/75), shaking chills (96%, 72/75), dizziness (75%, 56/75) and diarrhea (65%, 49/75) were also reported amongst study participants.

#### Mosquito vector behavior

The most commonly reported mosquito breeding sites were still-water and rubbish (71%, 53/75). Almost all caregivers (96%, 72/75) stated that malaria mosquitoes fed a night. When caregivers were asked what mosquitoes did after taking a blood meal (feeding), 36% (27/75) responded that fed mosquitoes land and rest on walls and roofs of the house with 11% (8/75) reporting fed mosquitoes left the house. The use of ITNs was 6% lower among caregivers who responded that mosquitoes landed on walls after feeding and 12% lower among those who stated that mosquitoes left the house after feeding.

#### Preventative strategies

All study participants responded sleeping under a mosquito net as a means of preventing malaria infection from mosquito bites (Table 3), with 97% (73/75) of caregivers also reported spraying insecticides on the walls of houses as a malaria prevention strategy. Other strategies mentioned included making fire and smoke (61%, 46/75), drinking lots of water (32%, 8/75) and eating garlic (27%, 20/75).

**Table 3:** Caregiver^a^ (participant) reported knowledge of malaria interventions in Luangwa and Nyimba Districts during the study period

	Nyimba n (%)	Luangwa n (%)	Total n (%)
Which of these are ways to prevent malaria mosquitoes from causing malaria?			
Sleeping under mosquito nets	40(100)	35 (100)	75 (100)
Eating garlic	15 (37.5)	5 (14)	20 (27)
Spraying insecticide on house walls	40 (100)	33 (94)	73 (97)
Making fire and smoke	24 (60)	22 (63)	46 (61)
Drinking lots of water	16 (40)	8 (23)	24 (32)
Other	1 (3)	0 (0)	1 (1)
How does spraying walls with insecticides prevent malaria for those living in the home?			
Prevents mosquitoes from resting on walls	18 (45)	8 (23)	26 (35)
Kills mosquitoes that land on the walls	24 (60)	25 (71)	49 (65)
Cleans the walls	1 (3)	1 (3)	2 (3)
Don’t know/Missing	1 (3)	0 (0)	1 (1)
Is there a fee to you when insecticides are sprayed on walls?			
Yes	3 (8)	3 (9)	6 (8)
No	34 (85)	30 (86)	64 (85)
Don’t know/Missing	3 (8)	2 (6)	5 (7)
How do treated mosquito nets prevent malaria for those who sleep under them?			
Prevent mosquitoes from biting	34 (85)	28 (80)	62 (83)
Kills mosquitoes that land on them	12 (30)	10 (29)	22 (29)
keeps people warm	1 (3)	1 (3)	2 (3)
Don’t know/Missing	1 (3)	1 93)	2 (3)
How does a spatial repellent prevent malaria for those people living in the home?			
Prevent mosquitoes from entering home	16 (40)	11 (31)	27 (36)
Kills mosquitoes that land on them	11 (28)	4 (11)	15 (20)
Don’t know	18 (45)	18 (51)	36 (48)
Is the amount of insecticides that are used in IRS and mosquito nets safe for you and your family?			
Yes	36 (90)	31 (89)	67 (89)
No	2 (5)	3 (9)	5 (7)
Don’t know/Missing	1 (3)	1 (3)	2 (3)

^a^woman > 18 years in charge of family care and household maintenance

Percentages may not sum to 100% because of multiple responses

All but one caregiver reported ever hearing of IRS. Sixty-five percent (49/75) of caregivers reported that IRS prevents malaria by killing mosquitoes that land on sprayed walls, while 35% (26/75) reported that IRS prevents malaria by preventing mosquitoes from resting on sprayed walls. When asked about costs of spraying IRS on their walls, most participants (85%, 64/75) stated that there was no fee to them for IRS applications. When asked how mosquito nets prevent malaria infection, 83% (62/75) of caregivers responded preventing mosquitoes from biting those under the mosquito net. With the choice of multiple responses, 29% (22/75) responded that mosquito nets prevent malaria by killing mosquitoes that land on them. Most caregivers (89%, 67/75) responded that the amount of insecticides used in IRS and mosquito nets was safe for them and their families.

Concerning spatial repellent product examples, such as mosquito coils, participants answered that spatial repellents may prevent malaria infection by preventing mosquitoes from entering the house (36%, 27/75) or killing mosquitoes that land on them (20%, 15/75). About half (48%, 36/75) of the participants reported to not knowing how spatial repellents prevented malaria infection.

### Attitudes towards malaria, interventions and barriers to uptake

**Fig. 5:**
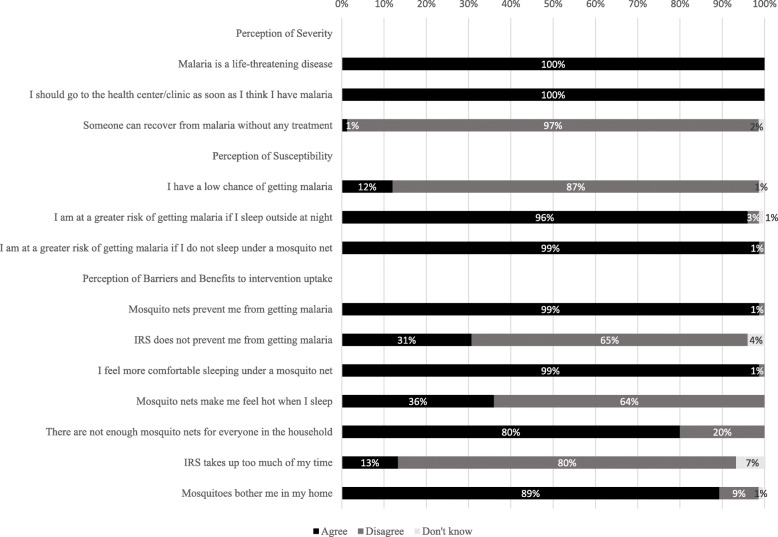
Caregiver stated attitudes towards malaria disease and interventions

Caregivers were asked questions related to their perceived susceptibility and severity of malaria. All caregivers agreed that malaria is a life-threatening disease and they should go to the health center/clinic as soon as they think they have malaria (Fig. 5). Only one caregiver reported that recovery from malaria occurs without any treatment and 12% (9/75) reported to having a low chance of getting malaria. Most participants agreed that they were at greater risk of getting malaria if they slept outside at night (96%, 72/75) and if they did not sleep under a mosquito net (99%, 74/75).

All but one caregiver reported that mosquito nets prevented them from getting malaria, and the majority of survey respondents (89%, 67/75) reported that mosquitoes bothered them in their home. When asked about household coverage, 80% (60/75) stated that there were not enough mosquito nets for everyone in their household. Most participants (99%, 74/75) answered that they felt comfortable sleeping under a mosquito net, although 36% (27/75) reported that mosquito nets made them feel hot. Thirteen percent of caregivers (10/75) reported that IRS application took too much of their time and 31% (23/75) agreed with the statement that IRS does not prevent them from getting malaria.

### Practices related to malaria interventions

In both districts, self-reported mosquito net use (sleeping under a mosquito net the night before the survey) was 67% (50/75) (Table 4). Sleeping under a mosquito net (79%, 59/75), clearing vegetation around the house (51%, 38/75) and draining still water (26%, 26/75) were the most commonly reported means of personal protection against malaria infection. Participants preferred this method of protection (nets) because: the method was better at stopping mosquito bites (76%, 57/75), nets are free (13%, 10/75), and nets are easy to use (15%, 11/75).

**Table 4:** Caregiver^a^ (participant) reported practices related to malaria

Practices related to malaria	Number	Percent
Did you sleep under a mosquito net last night?		
Yes	50	66.7
No	25	33.3
What personal protection measures do you currently practice to prevent malaria?		
Sleeping under mosquito nets	59	78.7
Clearing vegetation around the house	38	50.7
Draining still water	26	34.7
Wearing long-sleeves shirts and trouser/skirts	13	17.3
Spraying insecticides	7	9.3
Burning mosquito coil	3	4.0
Burning cow dung/leaves	2	2.7
Do nothing	8	10.7
Other	13	17.3
Which protection measure do you prefer the most?		
Burning mosquito coil	1	1.3
Sleeping under mosquito nets	61	81.3
Clearing vegetation around the house	3	4.0
Spraying insecticides	4	5.3
Don’t know/missing	4	5.3
None	1	1.3
Other	4	5.3
Why do you prefer practicing this protection technique?		
Free	10	13.3
Cheap	5	6.7
Better at stopping mosquitoes	57	76
Smells nice	4	5.3
Like the way it looks	3	4.0
Easy to use	11	14.7
Don’t know/missing	2	2.7
Other	12	16
If you wash your mosquito nets, do you use detergents when washing them?		
Yes	65	86.7
No	2	2.7
How do you dry the mosquito nets after washing?		
In the sunlight	42	56
In the shade	24	32
Don’t know/missing	7	9.3
Not applicable	2	2.7
How often do you tuck-in your mosquito net when you go to bed?		
Always (every night)	55	73.3
Sometimes (not every night)	11	14.7
Never	2	2.7
Don’t know/missing/NA	7	9.4
What time do you normally go to bed?		
Between dusk and 8 pm	24	32
Between 8 pm and 10 pm	42	56
Between 10 pm and midnight	5	6.7
After midnight	0	0
Don’t know/missing	4	5.3
Where do you normally cook?		
Inside the house	8	10.7
Outside the house (no shelter)	21	28
In a shelter closed on all four sides	40	53.3
In a cooking shelter not closed on all four sides	5	6.7
Other	1	1.3
Do you sleep outside the house?		
Yes (always)	1	1.3
Sometimes	40	53.3
No, never	34	45.3

^a^woman > 18 years in charge of rearing, family care and household maintenance

A majority (97%, 65/75) of caregivers who owned a mosquito net used some form of detergent in washing nets and over half (56%, 42/75) dried nets in the sunlight. When asked about sleeping practices, over half (56%, 42/75) of survey respondents answered that they normally went to bed between 8:00 PM and 10:00 PM and 32% (24/75) went to bed between dusk and 8:00 PM. About half (53%, 40/75) of the caregivers reported sometimes sleeping outside the house and 45% (34/75) reported never sleeping outside. Caregivers’ cooking practices included cooking in a shelter closed on all four sides (53%, 40/75), cooking outside the house in no shelter (28%, 21/75) and cooking inside the house (11%, 8/75).

### Acceptance of interventions

When asked about their mosquito nets, 63% (47/75) said they liked the color, 71% (53/75) liked the shape, and 85% (64/75) liked the size. About a three-quarters (72%, 54/75) of caregivers liked the “softness” of their mosquito nets. Concerning IRS application, 83% (62/75) of study participants reported the amount of time taken for IRS application was acceptable but the majority (57%, 43/75) reported not liking the smell of the insecticide. Almost all caregivers responded they liked the color (92%, 69/75), shape (96%, 72/75) and size (81%, 61/75) of the SR prototype with 88% (66/75) preferring the prototype to have had an odor, with the most commonly cited preferred smell to be of a ‘perfume’ (64%, 48/75).

### Entomological relationships with malaria KAP

A total of 73 participants (households) gave consent for indoor CDC mosquito trap collections. Overall, 398 mosquitoes were captured from 73 nights of collections: 49 anophelines and 349 culicines (Table 5). In Nyimba, a total of 50 mosquitoes were captured from 40 households (1.25 per household) compared to 348 mosquitoes from 33 households (10.5 per household) in Luangwa. *Anopheles arabiensis* represented the majority of all anophelines collected (39%; 19/49) from all households. Other species included: *An. funestus, An. parensis, An. quadriannulatus, An. rivulorum* and *An. squamosus,.* Of the anopheline mosquitoes, 12 were blood-fed with two *An. arabiensis* indicating feeding on humans. Other blood meal sources included goat (3 *An. quadriannulatus,* 2 *An. squamosus,* one *An. rivulorum,* one *An. parensis,* and one *An. arabiensis*) and cow (1 *An. rivulorum*)*.* One *An. squamosus* had both human and goat blood. *Plasmodium falciparum* circumsporozoites were indicated in one *An. arabiensis*.

**Table 5:** Total number of mosquitoes captured inside participant homes in Nyimba and Luangwa Districts, May–June 2016

	Nyimba District		Luangwa District		Both districts	
Mosquito type	Total No. mosquitoes^a^	Households surveyed n (%)	Total No. mosquitoes	Households surveyed n (%)	Total No. mosquitoes	Households surveyed n (%)
*An. funestus*	1	1 (3)	1	1 (3)	2	2 (3)
*An. arabiensis*	9	5 (13)	10	6 (18)	19	11 (15)
*An. squamosus*	1	1 (3)	11	4 (12)	12	5 (7)
*An. pretoriensis*	1	1 (3)	1	0 (0)	2	1 (1)
*An. rivulorum*	1	1 (3)	7	3 (9)	8	4 (5)
*An. quadriannulatus*	0	0 (0)	5	4 (12)	5	4 (5)
*An. parensis*	0	0 (0)	1	1 (3)	1	1 (1)
*Total anophelines*	13	9 (23)	36	18 (54)	49	27 (37)
*Total culicines*	37	9 (23)	312	23 (70)	349	32 (70)

^a^ using CDC miniature light traps placed at foot of bed and operating from 1800 h–0600 h on a single night

The percentage of caregivers who reported to use an ITN and were in households where at least one anopheline or culicine mosquito was captured, was higher in Luangwa (81%; 21/26) than in Nyimba (64%; 9/14). A similar trend was observed for IRS where the percentage of caregivers who reported to accept IRS and were in households where at least one anopheline or culicine mosquito was captured, was higher in Luangwa (96%; 25/26) than in Nyimba (92%; 13/14).

## Discussion

The uptake and use of currently recommended malaria interventions with demonstrated protective effects, such as IRS and ITNs, are critical for sustaining malaria control and mitigating malaria disease resurgence [29]. Likewise, in order for new vector control interventions, including those under development such as spatial repellents, to be viable complementary tools towards malaria elimination, these products must be accepted in endemic communities. The objective of this study was to assess malaria KAP of a primary caregiver cohort in Luangwa and Nyimba districts, Zambia to provide considerations to future educational campaigns.

Findings from this study indicate that most study participants had good knowledge of malaria disease, transmission routes and prevention strategies. The most commonly reported sources of malaria information were health clinics/centers and community health-workers, similar to other studies elsewhere in Zambia [47], reflecting positive outreach of these educational efforts. However, despite high malaria knowledge, misconceptions seem to persist within the study communities. Although most caregivers correctly cited the bite of an infected mosquito as a malaria transmission route, drinking dirty water and eating contaminated food were also reported by some individuals. These findings reflect outcomes from a study in Choma District, Zambia, which found that 1 in 10 study participants linked drinking bad water with malaria and 1 in 5 associated dirty surroundings with malaria [21]. These misconceptions could be due to misunderstandings between cholera and malaria transmission, two diseases that are of priority in education campaigns. Several caregivers in this study reported drinking chlorinated water, or placing chlorine in water used to wash ITNs, as one of their current malaria prevention strategies. This suggests that the caregivers surveyed may not clearly understand the distinction between cholera and malaria; a note being that such a misconception was derived from field notes and discussions with some of the study participants rather than directly by survey tools. In addition, the local Chichewa translation of the word *malaria* is *malungo,* which is ambiguously used to describe various febrile illnesses, especially those resulting in joint pain. The study team found that the term *malungo* was not recognized in certain communities, especially in Luangwa. A study conducted in Malawi found that the exact term, *malungo,* was used to refer to different diseases, including diarrhea, STDs, AIDS and malaria [33]. The use of different local words for malaria within the community may be contributing to misconceptions about malaria transmission, therefore sensitization campaigns and future KAP surveys should use the most culturally and community-specific appropriate terms when referring to malaria.

Attitudes related to malaria disease and interventions among caregivers were generally positive. Perception of disease severity was high, with all caregivers seeing malaria as a life-threatening disease and the majority perceiving susceptibility to infection. However, perceptions of benefits of ITNs were reported to be more favorable among caregivers than benefits of IRS. Eastern Province, Zambia, historically has received the highest IRS rates and Lusaka Province the lowest [3]. Thus, it was not surprising that the current study reported 2015/2016 IRS coverage higher in Nyimba (98%) district located in Eastern Province than in Luangwa (70%) district located in Lusaka Province. The authors note that caregiver responses in this study may not be an accurate reflection of actual IRS coverage as the questionnaire assessed whether the household had ever been sprayed with IRS rather than when the most recent spraying had occurred. In addition, not all households in the district were surveyed and, furthermore, participant recall bias may have also misrepresented IRS coverage. Despite this, almost all caregivers surveyed in both districts agreed that they would allow the application of IRS by spray teams when next scheduled. Interestingly, IRS acceptability proved to be high in both districts despite low levels of perceived efficacy. Similar findings were observed in neighboring Mozambique [22].

High IRS acceptance in spite of perception of low efficacy against malaria and concerns about the smell of the chemicals, noted by caregivers, may be due to other perceived benefits of IRS such as reduction of other insects like cockroaches in the house. A study conducted in Chiapas, Mexico found that certain members of the community were more likely to accept IRS because they perceived it to be effective in reducing the number of cockroaches and rats [48]. However, unlike this study, other studies have noted perceived increases in cockroaches and bedbugs as a reason for reduced community acceptance of IRS [22]. Future KAP studies should examine why IRS acceptability may be high despite it being perceived as having a low efficacy. This is needed to identify determinants of acceptance and adherence as well as possible overriding factors that could trigger intervention rejection [22]. Regarding study participant responses on ITN use, most caregivers who reported not being satisfied with the color of their ITNs said they would prefer “not white” colors. The reason for this, as stated by several participants, was that white ITNs show dirt and require more frequent washing. In fact, several caregivers explained that they did not use their ITN the night before because it was dirty and needed washing. This is similar to other reports in Kenya, where dark-colored nets were preferred because the smoke from firewood used for cooking was reported to leave brightly colored nets dirty [49]. Caregivers surveyed in the current study who did not like the shape of their current ITNs said they would prefer conical shaped ITNs as they felt they were easier to hang than the more common rectangular-shaped ITNs. Overall, tailoring ITN product selection by campaign organizers to match aesthetic and practical preferences and cultural needs at the household level in endemic communities may enhance ITN use and sustainability in Luangwa and Nyimba districts.

In order to relate malaria KAP with entomological trends, limited mosquito sampling was conducted during the study period. Trap collections demonstrated higher indoor mosquito density in Luangwa study households as those households in Nyimba, although overall anopheline capture was low. The species composition of mosquitoes captured in this study was quite varied with *An. arabiensis* and *An. squamosus* being the dominant species. More zoophagic than anthropophagic mosquitoes were captured indoors. The only species captured with human blood was *An. arabiensis.* It is noted that at the time of the study 98% of structures in Nyimba had been sprayed by IRS (through resources provided the U.S. President’s Malaria Initiative (PMI)) while in Luangwa, only 70% of households had been sprayed by the Ministry of Health. In Nyimba where IRS coverage is higher and more frequent, caregivers may have a higher perception of benefits and disadvantages of IRS than caregivers in Luangwa. It should be noted, however, that at the household level, IRS acceptability may not always be related to total number of mosquitoes in the home. For example, one participant in Nyimba reported that she had never allowed, and in the future will not allow, district teams to spray her house because there were no mosquitoes in the household. Interestingly, of the households sampled for mosquitoes in the current study in Nyimba, this household had the highest total number of indoor mosquitoes (21 mosquitoes) captured in traps. Additionally, the caregiver reported doing nothing to prevent malaria in her household. These findings highlight the importance of characterizing perceptions of community members to ensure appropriate messaging on various advantages of IRS that could be emphasized during IRS and educational campaigns in order to increase and sustain acceptability of this proven intervention [50].

The authors also recognize that mosquito collections at participant households may have been affected by ITNs provided by National Malaria Control Centre staff to protect participants from potential mosquito bites as a result of light traps being placed in the sleeping space. The insecticides on these new ITNs may have negatively impacted the total number of mosquitoes in the trapping space. Also, because collections were conducted between May and June, during the dry season, mosquito density and species may have been lower than if collections were performed earlier in the year. This is especially true within Nyimba where vector habitat is largely dependent on precipitation whereas Luangwa, located between the Luangwa and Zambezi Rivers, has more stable mosquito habitats.

Surveying caregiver acceptance and preferences of the SR prototype product revealed a high rate of potential uptake in this study area. Most participants in the current study preferred that the fully developed SR product have a “pleasant” smell, similar to findings from rural Tanzania which indicated the most important factors for community acceptance of topical repellents was providing the product with a pleasant smell, with a perceived irritating odor leading to reduced use among community members [8]. Several participants raised concerns about the ability to monitor the SR duration of efficacy (i.e. having an expiration indicator). Although interventions that do not require daily compliance may initially have a high community uptake [8], failure to communicate when such products should be replaced may reduce user compliance and efficacy of the intervention over time. Specifically, if communities expect the repellent effect to last longer than the product’s manufactured duration [51], the product may be perceived as ineffective and thus lead to low acceptance and/or compliance which will in turn render the product ineffective. A participatory formative approach such as Trials of Improved Practices (TIPs) could be used to assess community needs and preferences of new products, such as spatial repellents, on a small scale before introducing them more broadly [52]. Other important factors, not considered in this study, such as perception of harmful effects [53], willingness to pay [51] and introduction mechanisms, should also be explored in future studies assessing attitudes towards SR products.

The authors note study limitations to include study sample size and bias in self-reporting that precludes responses to be broadly representative of primary caregivers in Nyimba and Luangwa districts. It should be noted that study members found no direct translation of ‘spatial repellents’ in the Chichewa dialect therefore study members used common examples instead of an actual definition for the product. For example, spatial repellents were replaced with mosquito coils that may have biased results. However, with the SR prototype available for caregivers to touch and study team members explaining how the product is intended to be applied in the home such bias was mitigated. The study would have benefited from a qualitative probing through in-depth interviews (IDI) and/or focus group discussions (FGD) to further elucidate themes arising from the study. Thus, we recommend that future studies incorporate a robust qualitative component to their KAP studies. Despite these limitations, findings do provide initial insight on malaria KAP where previous studies have not been performed, and thus can guide robust KAP studies in the future. Perhaps most importantly, this study revealed important trends that may influence malaria prevention practices in these locations to be considered by local malaria control and elimination programs.

## Conclusion

Misconceptions about malaria transmission routes are critical to identify in endemic communities in order to facilitate successful disease and vector control. Findings from the current study indicate that the majority of participants correctly identified the route of malaria transmission to be mosquito-borne, but knowledge gaps were reported. This primary outcome supports the recommendation by the 2015 Zambian MIS that educational campaigns should focus on providing detailed information regarding the connection between mosquito bites and malaria parasite transmission to encourage ITN use and IRS acceptance. These messages in educational campaigns should be customized to the target populations with special attention to local terminology to reduce the chances of misconception or misinformation among community members. Providing CHWs, district health workers or volunteers with pre-defined scripts that fit community-specific knowledge gaps should be a consideration based on pre-KAP surveys, when possible. Likewise, though our findings indicate perceptions about malaria may be positive in the study communities, and both ITN and IRS are accepted strategies, a better understanding of underlying reasons for potential misalignment between presence of mosquitoes and perception of malaria risk in a household should be further explored and integrated into malaria control and elimination program efforts to facilitate intervention sustainability and compliance.

## Supplementary information

**Additional file 1.** KAP survey used to assess caregiver KAP in the study in Engligh and Nyanja.

## Data Availability

The datasets and other material analyzed during the current study are available from the corresponding author on reasonable request.

## Abbreviations

BCC: Behavior Change Communication
CHW: Community Health Worker
DDT: Dichlordiphenyltrichloroethane
IRS: Indoor Residual Spraying
ITN: Insecticide-treated nets
KAP: Knowledge, Attitudes and Practices
MIS: Malaria Indicator Survey
NMEP: National Malaria Elimination Programme
PE: Protective Efficacy
SR: Spatial Repellent
TIPs: Trials of Improved Practices
