# Supplementary material for: Knowledge, attitudes and practices assessment of malaria interventions in rural Zambia
Source: BMC Public Health. 2020 Feb 12;20:216. doi: 10.1186/s12889-020-8235-6 (PMC7017631; doi:10.1186/s12889-020-8235-6)
Supplement: Supplementary file 1 — Additional file 1. KAP survey used to assess caregiver KAP in the study in Engligh and Nyanja. [file 12889_2020_8235_MOESM1_ESM.docx]

# **Additional file 1**

## **KAP Questionnaire Form (English)**

**Assessment of Knowledge, Attitudes and Practices (KAP) of Malaria Interventions in Zambia**

**Full Survey**

Date: _________________________

Enumerator: ___________________

District: _______________________

Cluster number: ________________

**Specific Aim 1: Demographic factors and knowledge**

**Demographics:**

1. How old are you? (Tick only one box)
   - 18-20 (1)
   - 21-30 (1)
   - 31-40 (2)
   - 41-50 (2)
   - Above 50 (2)
2. What is the highest level of education of the head of the household? (Tick only one box)
   - No formal schooling (1)
   - Incomplete primary school (1)
   - Complete primary school (2)
   - Incomplete secondary school (2)
   - Complete secondary school (2)
   - Postsecondary and above (2)
3. What is the present occupation of the head of the household? _____________________________
4. How many people are permanent residents of this household? ____________________________
5. How many permanent household members are younger than 5 years of age? _________________
6. How many permanent household members are between the ages of 5 to 17 years of age? (adolescents)_______________
7. How many permanent household members are older than 18 years of age? __________________
8. How many permanent household members are currently pregnant? ________________________
9. Are there any mosquito nets in your household?
   - Yes (1) (9a) If yes how many? _____________
   - No (2)
10. Has your house been sprayed under the IRS programme by the district team?
    - Yes (1)
    - No (2)
11. Has the SHIELD been placed in your home?
    - Yes (1)
    - No (2)

**Basic Knowledge of malaria disease and mosquito behavior:**

If you don’t know the answer to any of these questions, feel free to say you don’t know.

1. Have you ever heard or received information about malaria?
   - Yes (1)
   - No (2)
   - Don’t know (3)
2. If yes, from which of these did you hear or receive information about malaria? (Tick all that apply)
   - Family member (at home) (1)
   - Neighbor (in the village) (2)
   - Radio (3)
   - Television (4)
   - Poster/information sheets (5)
   - Community Health Worker (6)
   - Health center/ clinic (7)
   - School (8)
   - Other (Specify)__________________________________________(9)
3. Which of these can give malaria to humans? (Tick only one box)
   - Rat (1)
   - Dog (2)
   - Mosquito (3)
   - Fly (4)
   - Cockroach (5)
   - Don’t know (6)
4. Malaria can be transmitted to humans by? (Tick all that apply)
   - Drinking dirty water (1)
   - Eating contaminated food (2)
   - Bite of a mosquito infected with malaria (3)
   - Touching a malaria patient (4)
   - Don’t know (5)
   - Other (describe)_______________________________________________________(6)
5. Which of these are malaria symptoms? (Tick all that apply)
   - High temperature/ Fever (1)
   - Vomiting (2)
   - swelling of feet (3)
   - Shaking chills (4)
   - Dizziness (5)
   - Itching (6)
   - Diarrhea (7)
   - Headache (8)
   - Don’t know (7)
   - Other (describe) ________________________________________________________(8)
6. Where do baby malaria mosquitoes live? (Tick all that apply)
   - Still water (1)
   - Rubbish (2)
   - Rice fields (3)
   - Rivers (4)
   - Forest pools (5)
   - Don’t know (6)
7. Do malaria mosquitoes feed at night? (Tick only one box)
   - Yes (1)
   - No (2)
   - Don’t know (3)
8. What do most mosquitoes usually do after they feed? (Tick only one box)
   - They die immediately (1)
   - They land and rest on the walls and roof of the house (2)
   - They leave the house (3)
   - Don’t know (4)

**Basic Knowledge relating to malaria vector control interventions:**

1. Which of these are ways to prevent malaria mosquitoes from causing malaria? (Tick all that apply)
   - Sleeping under mosquito nets (1)
   - Eating garlic (2)
   - Spraying insecticides on house walls (3)
   - Making fire and smoke (4)
   - Drinking lots of water (5)
   - Don’t know (6)
   - Others (describe) ______________________________________________________(7)
2. Have you ever heard of indoor residual spraying (IRS)? (Tick only one box) (If yes, conduct questions 23 and 24; if no, then move to question 25)
   - Yes (1)
   - No (2)
   - Don’t know (3)
3. How does spraying walls with insecticides prevent malaria for those people living in the home? (Tick all that apply)
   - Prevents mosquitoes from resting on the walls (1)
   - kills mosquitoes that land on the walls (2)
   - Cleans the walls (3)
   - Don’t know (4)
4. Is there a fee to you when insecticides are sprayed on walls? (Tick only one box)
   - Yes (1)
   - No (2)
   - Don’t know (3)
5. How do treated mosquito nets prevent malaria for those who sleep under them? (Tick all that apply)
   - Prevent mosquitoes from biting (1)
   - Kills mosquitoes that land on them (2)
   - Keeps people warm (3)
   - Don’t Know (4)
6. How does a spatial repellent prevent malaria for those people living in the home? (Tick all that apply)
   - prevent mosquitoes from entering the home (1)
   - Kills mosquitoes that lands on them (2)
   - Don’t know (3)
7. Is the amount of insecticides that are used in IRS and mosquito nets safe for you and your family? (Tick only one box) (if no, ask question 28; if yes, skip to question 29)
   - Yes (1)
   - No (2)
   - Don’t know (3)
8. Which strategies have insecticides that are not safe for you and your family? ___________________________________________________________

**Specific Aim 2: Cultural attitudes and practices**

**Cultural Attitudes towards malaria disease and interventions**

Tell me if you agree, disagree or are not sure about these statements.

1. Malaria is a life-threatening disease. (a)
   - Agree (1)
   - Disagree (2)
   - Not sure (3)
2. I should go to the health center/clinic as soon as I think I have malaria. (a)
   - Agree (1)
   - Disagree (2)
   - Not sure (3)
3. Someone can recover from malaria without any treatment. (a)
   - Agree (1)
   - Disagree (2)
   - Not sure (3)
4. I have a low chance of getting malaria. (b)
   - Agree (1)
   - Disagree (2)
   - Not sure (3)
5. I am at a greater risk of getting malaria if I sleep outside at night. (b)
   - Agree (1)
   - Disagree (2)
   - Not sure (3)
6. I am at a greater risk of getting malaria if I do not sleep under a mosquito net. (b)
   - Agree (1)
   - Disagree (2)
   - Not sure (3)
7. Mosquito nets prevent me from getting malaria. (c)
   - Agree (1)
   - Disagree (2)
   - Not sure (3)
8. IRS does not prevent me from getting malaria. (c)
   - Agree (1)
   - Disagree (2)
   - Not sure (3)
9. I feel more comfortable sleeping under a mosquito net. (c)
   - Agree (1)
   - Disagree (2)
   - Not sure (3)
10. Mosquito nets make me feel hot when I sleep. (e)
    - Agree (1)
    - Disagree (2)
    - Not sure (3)
11. There are not enough mosquito nets for everyone in the household. (e)
    - Agree (1)
    - Disagree (2)
    - Not sure (3)
12. IRS takes up too much of my time. (e)
    - Agree (1)
    - Disagree (2)
    - Not sure (3)
13. Mosquitoes bother you in your home.
    - Agree (1)
    - Disagree (2)
    - Not Sure (3)

**Practices and Uses related to malaria prevention**

1. What personal protection measures do you currently practice to prevent malaria? (Tick all that apply)
   - Burning mosquito coil (1) - if so, ask question 42.
   - Burning cow dung/leaves (2) - if so, ask question 43.
   - Sleeping under mosquito nets (3) - if so, ask question 44.
   - Wear long-sleeve shirt and trousers/skirts (4)
   - Draining still water (5)
   - Clearing vegetation around the house (6)
   - Spraying insecticides (7)
   - Do nothing (8)
   - Don’t know (9)
   - Others (Specify): ________________________________________________________(10)
2. How many times a week do you burn mosquito coils?
   - Seven times or more (1)
   - Four to six times (2)
   - One to three times (3)
   - Don’t know (4)
3. How many times a week do you burn cow dung/leaves?
   - Seven times or more (1)
   - Four to six times (2)
   - One to three times (3)
   - Don’t know (4)
4. Did you sleep under a mosquito net last night?
   - Yes (1)
   - No (2)
   - Don’t know (3)
5. Which protection measure do you prefer the most? (Tick only one box)
   - Burning mosquito coil (1)
   - Burning cow dung/leaves (2)
   - Sleeping under mosquito nets (3)
   - Wear long-sleeve shirt and trousers (4)
   - Draining still water (5)
   - Clearing vegetation around the house (6)
   - Spraying insecticides (7)
   - Don’t know (8)
   - None (9)
   - Others (Specify): ______________________________________________________(10)
6. Why do you prefer practicing this protection technique? (Tick all that apply)
   - Free (1)
   - Cheap (2)
   - Better a stopping mosquito bites (3)
   - Smells nice (4)
   - Like the way it looks (5)
   - Easy to use (6)
   - Don’t know (7)
   - Other (Specify): ________________________________________________________(8)
7. Who sleeps under the mosquito nets? (Tick all that apply)
   - All family members (1)
   - Father and mother (2)
   - Children under the age of 5 years (3) How many? __________________
   - Children between the ages of 5 and 17 years (4) How many? __________________
   - Nobody (5)
   - Don’t know (6)
   - Others (Specify): _______________________________________________________(7)
8. How often do you wash your mosquito nets? (Tick only one box)
   - Once in a month (1)
   - Once in six months (2)
   - Once a year (3)
   - Not washing (4)
   - Don’t know (5)
   - Not applicable (6)
9. If you wash you mosquito nets, do you use detergents when washing them? (Tick only one box)
   - Yes (1)
   - No (2)
   - Don’t know (3)
   - Not applicable (4)
10. How do you dry the mosquito nets after washing? (Tick only one box)
    - In the sunlight (1)
    - In the shade (2)
    - Don’t know (3)
    - Not applicable (4)
11. How often do you tuck-in your mosquito net when you go to bed? (Tick only one box)
    - Always (every night) (1)
    - Sometimes (not every night) (2)
    - Never (3)
    - Don’t know (4)
    - Not applicable (5)
12. What time do you normally go to bed? (Tick only one box)
    - Between dusk and 8pm (1)
    - Between 8pm and 10pm (2)
    - Between 10pmand midnight (3)
    - After midnight (4)
    - Don’t know (5)
13. Where do you normally cook? (Tick only one box)
    - Inside the house (1)
    - Outside the house (no shelter) (2)
    - In a shelter closed on all four sides (3)
    - In a cooking shelter not closed on all four sides (4)
    - Don’t know (5)
    - Other (Specify) __________________________________________________ (6)
14. Do you sleep outside the household? (Tick only one box)
    - Yes, always (1)
    - Sometimes (2)
    - No, never (3)
    - Don’t know (4)

**Acceptability of malaria vector control interventions**

**Mosquito nets - ask only if use and/or in household**

1. Do you like the color of your mosquito net? (Tick only one box)
   - Yes (1)
   - No (2), if no what color would you prefer? ___________________________
   - Don’t know (3)
2. Do you like the shape or your mosquito net? (Tick only one box)
   - Yes (1)
   - No (2), if no what shape would you prefer? __________________________
   - Don’t know (3)
3. Do you like the size of mosquito net? (Tick only one box)
   - Yes (1)
   - No (2), if no would you prefer bigger or smaller nets? __________________
   - Don’t know (3)
4. Do you like the softness of your mosquito net? (Tick only one box)
   - Yes (1)
   - No (2)
   - Don’t know (3)
5. If the bed nets you own were not given to you free, would you still buy them? (Tick only one box)
   - Yes (1)
   - No (2)
   - Maybe (3)
   - Don’t know (4)
6. Would you recommend mosquito nets to a friend or family member who is not using or does not have a mosquito net? (Tick only one box)
   - yes (1)
   - no (2)
   - maybe (3)
   - Don’t know (4)

**IRS - ask of all participants**

1. In the future will you allow or continue to allow the spraying of insecticides on your walls?
   - Yes (1)
   - No (2)
   - Maybe (3)
   - Don’t know (3)

(For all answers) Why? ___________________________________________________________

1. If your house has been sprayed with insecticide before, did you like the smell of the insecticide?
   - Yes (1)
   - No (2)
   - Don’t know (3)
   - Not applicable (4)
2. If your house has been sprayed with insecticide before, were you fine with the amount of time it took?
   - Yes (1)
   - No (2)
   - Don’t know (3)
   - Not applicable (4)

**SHIELD - ask only if they answer yes to question 11**

1. Do you like the color of your SHIELD?
   - Yes (1)
   - No (2), if no what color would you prefer? ___________________________
   - Don’t know (3)
2. Do you like the shape of your SHIELD?
   - Yes (1)
   - No (2), if no what shape would you prefer? __________________________
   - Don’t know (3)
3. Do you like the size of your SHIELD?
   - Yes (1)
   - No (2), if no would you like it bigger or smaller? ______________________
   - Don’t know (3)
4. If not given to you free in the future, would you buy the SHIELD?
   - Yes (1)
   - No (2)
   - Maybe (3)
   - Don’t know (4)

(For all answers) Why? ____________________________________________________________________________________________________________________________________________________________

1. Would you prefer your SHIELD to have a smell?
   - Yes (1), if yes what smell would you prefer? __________________________
   - No (2)
   - Don’t know (3)
2. Do you think the SHIELD is safe for your family?
   - Yes (1)
   - No (2)
   - Maybe (3)
   - Don’t know (4)
3. Would you recommend SHIELD to other family members and friends?
   - Yes (1)
   - No (2)
   - Maybe (3)
   - Don’t know (4)

(For all answers) Why? ____________________________________________________________________________________________________________________________________________________________

**KAP Questionnaire Form (Nyanja)**

**Kuonapo Pa Chidziwitso, Khalidwe Ndi Mchitidwe Zokhudzana Ndi Matenda A Malungo Mdziko La Zambia.**

Date;……………………………………………………………………..

Enumerator;…………………………………………………………………………………………………

District;…………………………………………………………………………………………………………

Cluster;………………………………………………………..

**Chiyambi, 1. Za Pakhomo Pano**

1. Kodi muli ndi zaka zingati?

- 18-20 (1)
- 21-30 ( 1)
- 31-40 (2)
- 41-50 (2)
- Kupitilira 50 (2)

1. Kodi mutu wapano pa nyunba anaphunzira kufikira motani?

- Sanaphunzirepo yayi (1)
- Anaphunzira koma sanatsirize primary (1)
- Anaphunzira ndipo anatsiriza primary chabe (2)
- Anaphunzira ndipo sanatsirize secondary (2)
- Anaphunzira ndipo anatsiriza secondary chabe (2)
- Anaphunzira kwambiri koposa secondary (2)

1. Kodi mutu wapanyumba pano ntchito yeni-yeni agwira ndi yotani?
2. Kodi ndi angati amene ali nzika za pa nyumba zapano?
3. Kodi ndi angati amene akalibe ufikitsa zaka 5 pa nzika za pano pa nyumba?
4. Kodi ndi angati amene ali ndi zaka pakati pa 5 ndi 17?
5. Kodi ndi angati amene ali ndi zaka zopitilira pa 18 pa nzika za pano pa nyumba?
6. Kodi pa nzika za pano pa nyumba ndi angati azimai ali ndi pathupi?
7. Kodi pano pa nyumba muli ndi maukonde otetezera ku matenda ya malungo?

- inde (1) (9a) kodi ndi yangati?
- Ayi (2)

1. Kodi anabwerapo pano pa nyumba a zaumoyo waku boma kudzafafaza mnyumba mankhwala yophera udzudzu?

- Inde (1)
- Ayi (2)

1. Kodi mnyumba mwanu munaikidwa SHIELD?

- Inde (1)
- Ayi (2)

**Kadziwidwe pa za matenda ya malungo ndi zichitidwe za udzudzu:**

Ngati simunadziwe yankho iliyonse pa mafunso ali munsimu mukhale omasuka kunena kuti sindidziwa.

1. Kodi manamvapo olo kulandilapo uthenga ulionse pa za matenda ya malungo?

- Inde (1)
- Ayi (1)
- Sindidziwa (3)

1. Ngati munamvapo za uthenga umeneu kodi mnamva kwa ndani?

- Mmodzi mwa achibale pano pakhomo (1)
- Ku anthu a mmudzi muno (2)
- Pa wailes (3)
- Pa wailesi ya kanema (4)
- Pa chikwangwanu (5)
- Kwa othandizira za umoyowa mmudzi (chw) (6)
- Ku chipatala (7)
- Ku sukulu (8)
- Kwina kwache……………………………………………………(9)

1. Kodi ndi chotani pa izi zili munsimu zimene zingathe kudzesa matenda a malungo ku anthu?

- Khoswe (1)
- Galu (2)
- Udzudzu (3)
- Nchenche (4)
- Mphenvu (5)
- Sindidziwa (6)

1. Matenda ya malungo yangapatsidwe ku anthu mnjira ya?

- Kumwa madzi yosasamalika/ ya dothi (1)
- Kudya zakudya zilibe ukhondo (2)
- Ulumiwa ndi udzudzu umene umapatsa matenda ya malungo (3)
- Ugwira munthu amene adwala matenda ya malungo (4)
- Sindidziwa (5)
- Kwina kwacha……………………………………………………………………….. (6)

1. Kodi zizindikiro zakudwala matenda ya malungo ndi zotani pa izi?

- Kuphya thupi (1)
- Usanza (2)
- Utupa mapazi (3)
- Ugwedezeka kwa chigama (4)
- Chidima (5)
- Kunyeleza (6)
- Mmimba mothulula (7)
- Kuwawa mutu (8)
- Sindidziwa (9)
- Zina zilizonse……………………………………………(10)

1. Kodi tu udzudzu tung’ono-tung’ono tumene tumapatsa matenda ya malungo tukhala kuti?

- Mmadzi yokhazikika (1)
- Kuzinyalala (2)
- Mmunda wa mpunga (3)
- Mmitsinje (4)
- Mnkhalango (5)
- Sindidziwa (6)

1. Kodi udzudzu umene upatsa matenda ya malungo umadya usiku?

- Inde (1)
- Ayi (2)
- Sindidziwa (3)

1. Kambiri kodi udzudzu wambiri umachita zotani ukatsiriza kudya?

- Umafa pamenepo (1)
- Umakhazikika ndi kupumula mzipupa ndi ku tswindi ya nyumba (2)
- Umachoka pa nyumba (3)
- Sindidziwa (4)

**Kadziwidwe kulingana ndi kufuna-funa pa za kathetsedwe ka matenda ya malungo.**

1. Kodi niziti mwai izi zili mmunsimu zimene zithandiza kupewetsa udzudzu umene ubweretsa matenda ya malungo kuti upatse matenda ya malungo?

- Ugona mu ukonde (1)
- Kudya garlic (2)
- Kufafaza mankhwala ophera tudoyo kuzipupa kwa nyumba (3)
- Usonkha moto ndi kupukiza (4)
- Kumwa madzi ambiri kwambiri (5)
- Sindidziwa (6)
- Zina zailizonse (7)

1. Kodi munanvapo za indoor residue spraying(**IRS**) kufafaza mankhwala mmanyumba kufuna kuteteza udzudzu? (chongani pa modzi chabe)(ngati anamvapo,funsani mafunso ya chinambala 23 ndi 24;koma ngati sanamvepo,lumphani mufunse funso yachinambala 25)

- Inde (1)
- Ayi (2)
- Sindidziwa (3)

1. Kodi kufafaza mankhwala ophera tudoyo mzipupa mwa nyumba kuthandiza bwanji kupewa matenda ya malungo ku anthu amene akhala mnyumbamu?

- Kuletsa udzudzu ukhala ku zipupa (1)
- Kukupha udzdudzu umene utsikira mzipupa (2)
- Kuyeletsa zipupa (3)
- Sindi dziwa (4)

1. Kodi amakulipilitsani akakufafazirani mankhwala ophera tudoyo kuzipupa za nyumba yanu?

- Inde (1)
- Ayi (2)
- Sindidziwa (3)

1. Kodi ukonde oikidwa mankhwala kuteteza matenda ya malungu uthandizira bwanji kwa anthu amene agonamo?

- Uteteza kulumidwa ndi udzudzu (1)
- Ukupha udzudzu ukango khalako (2)
- Ubweretsa thumira ku anthu (3)
- Sindidziwa (4)

1. Kodi mankhwala ofafaza mnyumba kuteteza tudoyo yathandiza bwanji kupewa matenda ya malungo ku anthu okhala mnyumbamo?

- Yaletsa udzudzu kulowa mnyumba (1)
- yakupha udzudzu ukakhalapo (2)
- sindidziwa (3)

1. Kodi muyeso wamankhwala ophera tudoyo umene usewenzetsedwa ndi **IRS** komanso ukonde ,ulibwino motani kwa inu ndi banja lanu? (chongani pamodzi chabe)(ngati akana,funsani funso la nambala 28; ngati inde,funsani funso 29)

- Inde (1)
- Ayi (2)
- Sindidziwa (3)

1. Kodi ndi njira zotani za mankhwala ophera tudoyo zimene sizili bwino kwa inu ndi banja lanu?

……………………………………………………………………………………………………………………………………………….

**Cholinga chachiwiri; chikhalidwe chathu ndi mchitidwe.**

**chikhalidwe chokhudza pa matenda a malungo ndi ndondomeko zofuna kuteteza.**

Mund uze ngati mubvomeleza, simubvomeleza olo kaya pamakambidwe ali munsimu.

1. Matenda ya malungo ndi matenda amene ayophyeza umoyo wa munthu. (a)

- Ndibvomera (1)
- Sindibvomera (2)
- Kaya (3)

1. Nkofunikira kupita ku chipatala msanga ndikaganiza kuti ndili ndi matenda ya malungo. (a)

- Ndibvomera (1)
- Sindibvomera (2)
- Kaya (3)

1. Munthu angathe kupola kumatenda ya malungo kopanda kuchiritsidwa ndi china chilichonse. (a)

- Ndibvomera (1)
- Sindibvomera (2)
- Kaya (3)

1. Ndi chapatali kwa ine kudwala matenda ya malungo. (b)

- Ndibvomera (1)
- Sindibvomera (2)
- Kaya (3)

1. Ngati nagona pabwalo usiku ndichapafupi kwambiri kudwala matenda ya malungo. (b)

- Ndibvomera (1)
- Sindibvomera (2)
- Kaya (3)

1. Ngati sindigona mu ukonde nchapafupi kwaine kudwala matenda ya malungo. (b)

- Ndibvomera (1)
- Sindibvomera (2)
- Kaya (3)

1. Ukonde unditetezera kuti ndisatengeko matenda ya malungo. (c)

- Ndibvomera (1)
- Sindibvomera (2)
- Kaya (3)

1. **IRS** sikwanitsa kundichinjiliza ku matenda ya malungo. (c)

- Ndibvomera ( 1)
- Sindibvomera ( 2)
- Kaya ( 3)

1. Ndimvera bwino kwambiri ugona mu ukonde. (c)

- Ndibvomera (1)
- Sindibvomera (2)
- Kaya (3)

1. Nimamvera kuphya ngati nagona mu ukonde. (e)

- Nibvomera ( 1)
- Sindibvomera (2)
- Kaya (3)

1. Maukonde amene tili nayo pano pa nyumba siyakwanila, anthu ndife ambiri. (e)

- Ndibvomera (1)
- Sindibvomera (2)
- Kaya (3)

1. **IRS** imanditaila nthawi. (e)

- Ndibvomera (1)
- Sindibvomera (2)
- Kaya (3)

1. Udzdudzu umatisokoneza mnyumba mwathu.

- Ndibvomera (1)
- Sindibvomera (2)
- Kaya (3)

**Mchitidwe ndi zochitika pakuthandizira kupewa matenda ya malungo.**

1. Pa inu nokha, kodi mumachita zotani palipano zimene zithandizira kupewa matenda ya malungo? (chongani mayankho yonse yamene yayankhidwa)

- Kutentha ka koilo (coil) kophera udzudzu (1) ngati ndi inde,funsani funso 42.
- Kutentha tubvi twa ng’ombe/ mayani (2)ngati ndi inde, funsani funso 43.
- Kugona mu ukonde (3)ngati ndi inde, funsani funso 44.
- Kubvala Malaya yatali manja ndiponso mabuluku/ sikati (4)
- Kuchotsa madzi yokhalira (5)
- Kuseula pa khomo (6)
- Kufafaza mankhwala ophera tudoyo (7)
- Osacita chita chiriconse khale basi (8)
- Sindidziwa (9)
- Chinacilichonse……………………………………………………………………. (10)

1. Kodi mumatentha kangati ma koilo (coil) yophera udzudzu pa sondo limodzi?

- Kali 7 ndiku pyola (1)
- Pakati pa 4-6 (2)
- Pakati pa 1-3 (3)
- Sindidziwa (4)

1. Kodi mumatentha kangati matuvi ya ng’ombe/ mayani pa sondo limodzi?

- Kali 7 ndi kupyola (1)
- Pakati pa 4-6 (2)
- Pakati pa 1-3 (3)
- Sindidziwa (4)

1. Kodi munagona mu ukonde usiku watha?

- Inde (1)
- Ayi (2)
- Sindidziwa (3)

1. Kodi ndi njira ya chitetezo yotani imene imakukondweretsani kwambiri? (chongani yankho imodzi chabe)

- Kutentha ka koilo (coil) kophera udzudzu (1)
- Kutentha tubvi twa ng’ombe/ mayani (2)
- Kugona mu ukonde (3)
- Kubvala Malaya yatali manja ndima buluku (4)
- Kuchotsa madzi yokhalira (5)
- Kuseula pakhomo (6)
- Kufafaza mankhwala yophera tudoyo (7)
- Sindidziwa (8)
- Palibe (9)
- Chinachiliconse……………………………………………………………………………………….(10)

1. Kodi nchifukwa chiyani mukonda usewenzetsa njira yachitetezo imeneyi? Chongani yonse mayankho yamene ayankhidwa)

- Yamahala (1)
- Ndiyochipa (2)
- Ndiya bwino pakathetsedwe ka udzudzu (3)
- Yanunkhira bwino (4)
- Yaoneka bwino (5)
- Siyabvuta kusewenzetsa (6)
- Sindidziwa (7)
- Chinachiliconse……………………………………………………..(8)

1. Kodi ndani agona mu ukonde? (chongani mayankho yonse yamene ayankhidwa)

- Onse anthu apano pakhomo (1)
- Atate ndi amai (2)
- Ana amene sanakwanitse zaka 5 (3)
- Ana ali ndi zaka pakati pa 5-17 (4)
- Kulibe (5)
- Sindidziba (6)
- Ena ali onse……………………………………………………………………………………(7)

1. Kodi pamapita nthawi yaitali bwanji kuti muwache ukonde wanu? (chongani yankho imodzi chabe)

- Kamodzi pa mwezi (1)
- Kamodzi pa miyezi 6 (2)
- Kamodzi pa chaka (3)
- Sitimaiwacha (4)
- Sindidziwa (5)
- Kulibe (6)

1. Ngati mumauwacha ukonde wanu, kodi mumasewenzetsa sopo wa phala? (chongani yankho imodzi chabe)

- Inde (1)
- Ayi (2)
- Sindidziwa (3)
- Kulibe (4)

1. Kodi mumaumika mnjira yotani ukonde wanu mkauwacha? (chongani yankho imodzi chabe)

- Pa dzuwa (1)
- Pa mthunzi (2)
- Sindidziwa (3)
- Kulibe (4)

1. Kodi mumavwitikira nthawi zonse ukonde wanu mkapita ukagona kuchipinda? (chongani yankho imodzi chabe)

- Inde usiku ulionse (1)
- Inde timavwitikira koma osati usiku ulionse. (2)
- Yai (3)
- Sindidziwa (4)
- Kulibe (5)

1. Kodi mumapita nthawi zanji ukagona ku chipinda? (chongani yankho imodzi chabe)

- Pakati pa mmadzulo ndi 20hrs (8pm) (1)
- Pakati pa 20-22 hrs (2)
- Pakati pa 22hrs ndi pakati pa usiku (3)
- Upitilira pakati pa usiku (4)
- Sindidziwa (5)

1. Kodi mumaphikira kuti? (chongani yankho imodzi chabe)

- Mkati mwa nyumba (1)
- Pabwalo pa nyumba pamtetete (2)
- Mkakhicheni komata kapa bwalo (3)
- Mkakhumbi (4)
- Sindidziwa (5)
- Kwinakulikonse…………………………………………………………………………………(6)

1. Kodi mugona pabwalo pa nyumba yanu? (chongani yankho imodzi chabe)

- Inde nthawi zones (1)
- Inde nthawi zina (2)
- Ayi olo mpang’ono pomwe (3)
- Sindidziwa (4)

**Kalandilidwe ka njira zimene zinaikidwa zothetselamo matenda ya malungo.**

**Ukonde- Funsani chabe ngati asewenzetsa olo alinayo mnyumba.**

1. Kodi muukonda mtundu wa ukonde wanu? (chongani yankho imodzi chabe)

- Inde (1)
- Ayi (2)ngati ayi, kodi mungakonde mtundu wanji?...................................................
- Sindidziwa (3)

1. Kodi muyakonda mapangidwe ya ukonde wanu? (chongani yankho imodzi chabe)

- Inde (1)
- Ayi (2)ngati ayi, mukonda mapangidwe yotani?.....................
- Sindidziwa (3)

1. Kodi ndinu okondwa ndi ukulu wa ukonde wanu? (chongani yankho imodzi chabe)

- Inde (1)
- Ayi (2) ngati ayi, kodi mungafuni ukonde waukulu olo waung’ono?
- Sindidziwa (3)

1. Kodi ndinu okondwa pakafewedwe ka ukonde wanu? (chongani yankho imodzi chabe)

- Inde (1)
- Ayi (2)
- Sindidziwa (3)

1. Ngati ukonde amene mulinao simunapatsidwe mahala, kodi mungafune kuugura ukondeu? (chongani yankho imodzi chabe).

- Inde (1)
- Ayi (2)
- Kapena (3)
- Sindidziwa (4)

1. Kodi mungapatse uphungu kwa mzanu olo wachibale wamene alibe ukonde pa za ubwino wokhala ndi ukonde? (chongani yankho imodzi chabe)

- Inde (1)
- Ayi (2)
- Kapena (3)
- Sindidziwa (4)

**IRS – Funsani onse otengako mbali**

1. Kodi muzabvomekeza olo upitiliza kuwabvomekeza kuti zipupa zanu zizi fafazidwa ndi mankhwala ophera tudoyo?

- Inde (1)
- Ayi (2)
- Kapena (3)
- Sindidziwa (4)

(kuma yankho yonse) chifukwa chain?………………………………………………………………..

1. Ngati nyumba yanu ndi imodzi mwa zamene zinafafazidwapo mankhwala ophera tudoyo, kodi munakondwera nako kafungo ka mankhwala?

- Inde (1)
- Ayi (2)
- Sindidziwa (3)
- Kulibe (4)

1. Ngati nyumba yanu ndi imodzi mwazamene zinafafazidwapo mankhwala ophera tudoyo , kodi munakondwera nayo nthawi imene ntchitoyi inatenga kuti imalizike?

- Inde (1)
- Ayi (2)
- Sindidziwa (3)
- Kulibe (4)

**SHIELD – Funsani funsoli kwa aja okhao ayankha kuti inde ku funso ya nambala 11.**

1. Kodi muukonda mtundu wa **SHIELD** yanu?

- Inde (1)
- Ayi (2) ngati ayi,mungafune mtundu wanji?....................................................................
- Sindidziwa (3)

1. Kodi muyakonda maonekedwe ya **SHIELD** yanu?

- Inde (1)
- Ayi (2) ngati ayi, kodi mungafune maonekedwe otani?............................................................
- Sindidziwa (3)

1. Kodi ndinu okondwa ndi ukulu wa **SHIELD** yanu?

- Inde (1)
- Ayi (2) ngati ayi, kodi mungafune **SHIELD** yaikulu olo yaing’ono?
- Sindidziwa (3)

1. Ngati simunapatsidwe ya mahala mtsogolo, kodi mungagule **SHIELD**?

- Inde (1)
- Ayi (2)
- Kapena (3)
- Sindidziwa (4)

(kumayankho yonse) chifukwa chain?........................................................................................

……………………………………………………………………………………………………………………………………………

1. Kodi mungafune kuti **SHIELD** yanu izinunkhira?

- Inde (1) ngati inde, mungafune kununkhira kotani?
- ayi (2)
- sindidziwa (3)

1. Kodi muganiza kuti **SHIELD** ndiya bwino ku banja lanu?

Inde (1)

Ayi (2)

Kapena (3)

Sindidziwa (4)

1. Kodi mungapatse uphungu wa **SHIELD** ku wa banja ndi anzanu?

- Inde (1)
- Ayi (2)
- Kapena (3)
- Sindidziwa (4)

(pa mayankho onse) kodi ndi chifukwa chotan?....................................................................................................................................................................................................................................................................................................................................................................................................................................................................
